# The Fibrillar Collagen Family

**DOI:** 10.3390/ijms11020407

**Published:** 2010-01-28

**Authors:** Jean-Yves Exposito, Ulrich Valcourt, Caroline Cluzel, Claire Lethias

**Affiliations:** Institut de Biologie et Chimie des Protéines, IFR 128 Biosciences Lyon-Gerland, CNRS UMR 5086, Université de Lyon, 7 passage du Vercors, F-69367 Lyon Cedex 07, France; E-Mails: u.valcourt@ibcp.fr (U.V.); caroline.cluzel-grangeasse@ibcp.fr (C.G.); c.lethias@ibcp.fr (C.L.)

**Keywords:** fibrillar collagen, extracellular matrix, metazoan evolution

## Abstract

Collagens, or more precisely collagen-based extracellular matrices, are often considered as a metazoan hallmark. Among the collagens, fibrillar collagens are present from sponges to humans, and are involved in the formation of the well-known striated fibrils. In this review we discuss the different steps in the evolution of this protein family, from the formation of an ancestral fibrillar collagen gene to the formation of different clades. Genomic data from the choanoflagellate (sister group of Metazoa) *Monosiga brevicollis*, and from diploblast animals, have suggested that the formation of an ancestral α chain occurred before the metazoan radiation. Phylogenetic studies have suggested an early emergence of the three clades that were first described in mammals. Hence the duplication events leading to the formation of the A, B and C clades occurred before the eumetazoan radiation. Another important event has been the two rounds of “whole genome duplication” leading to the amplification of fibrillar collagen gene numbers, and the importance of this diversification in developmental processes. We will also discuss some other aspects of fibrillar collagen evolution such as the development of the molecular mechanisms involved in the formation of procollagen molecules and of striated fibrils.

## 1. Introduction

Collagen, in all its forms, represents the most abundant protein in animals. The collagens represent a heterogeneous family of extracellular matrix glycoproteins containing at least one triple helical domain and are generally involved in the formation of supramolecular networks [1,2]. All of the collagen molecules are made up of three α chains that may or may not be identical. At the primary structure level, the sequence of α chains involved in the formation of triple-helical structure consists of repeating Gly-Xaa-Yaa triplets and is called the collagenous domain or triple helix motif. Thus, the triple-helical structure corresponds to a right-handed superhelix resulting from the intertwining of the collagenous domains of three α chains, each of which adopts a polyproline II-like left-handed conformation. Collagens are often considered as a metazoan hallmark, even if proteins possessing triple-helical motifs have been identified in viruses, bacteria, fungi and the protists choanoflagellates [2–5]. Moreover, some other metazoan proteins contain a triple helix but are not members of the collagen family. These include several humoral proteins included in the so-called defense collagen family, and implicated in innate immunity [2,6].

Among the 28 different types identified in vertebrates [7,8], basement membrane type IV and the fibrillar collagens are the only ones to have been hitherto described from sponges to humans [9,10]. Type IV collagen is one of the major constituents of basement membranes where it forms a three-dimensional network. It has been characterized in Homoscleromorpha, the only sponge group presenting basement membrane-like structures. Interestingly, in another sponge group devoid of basement membrane structure, the Demospongiae, a short-chain collagen family evolutionarily related to type IV collagens has been described. These short-chain collagens seem to be present only in invertebrates with the notable exception of Ecdyzozoa [11].

**Figure 1.**
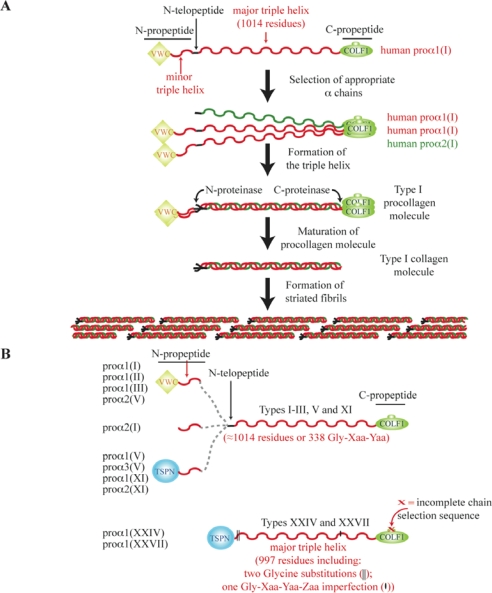

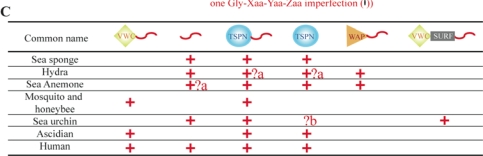
Fibrillar collagen characteristics. (A) From fibrillar α chains to the striated fibrils. To illustrate the different steps leading to the formation of collagen fibrils, we have chosen human type I collagen. After the selection of appropriate α chains, the *C*-propeptides play a fundamental role in alignment and registration of α chains, permitting nucleation of the triple helix and its elongation in a *C*- to a *N*-terminal direction. Then, the procollagens are processed by *N*- and *C-*proteinases, and the resultant collagen molecules aggregate to form the fibrils. (B) Modular structures of human fibrillar collagen chains. Dashed lines are used to illustrate the three different *N*-propeptide modular configurations found in human types I–III, V and XI. (C) Different *N*-propeptide architectures of metazoan fibrillar collagen chains. (+) indicates that at least one α chain presents this type of *N*-propeptide. In the case of cnidarian data, (+?a) signifies that these *N*-propeptide modular structures have not been characterized to date, but are present either in hydra or in sea anemone. (?b) indicates that phylogenetic analysis has indicated that the sea urchin 7α chain is related to vertebrate types XXIV and XXVII, but that the *N*-propeptide of this fibrillar collagen is still unknown [12].

The fibrillar collagens are present in almost all animals, and are the components of the well-known striated fibrils. A prototypal fibrillar procollagen α chain consists of an uninterrupted collagenous domain or major triple helix made up of approximately 338 Gly-Xaa-Yaa triplets, this region being flanked by two non-collagenous domains, the *N*- and the *C*-propeptides. On the biosynthetic pathway leading to the formation of the striated fibrils (Figure 1A), we first have the selection and association of three α procollagen chains. The resultant procollagen molecules are processed into collagen molecules. During this maturation, the *N*- and the *C*-propeptides are generally cleaved by specific proteases. The collagen molecules mostly correspond to the major triple helix and appear as rod-like structures 300 nm in length with diameter approximately 1.5 nm. Two short non-collagenous segments or telopeptides flank the major triple helix in these mature collagen molecules, which are then able to assemble into fibrils. The triple helical structure is less susceptible to proteases than non-collagenous domains, and since fibrillar collagens are abundant proteins, this might explain why they have been useful in analyzing palaeontologic material [13,14]. Indeed, taking advantage of the fact that vertebrate type I collagen is one of the most abundant vertebrate proteins, several studies have been able to sequence part of this well preserved molecule using old fossilized bones from dinosaurs 68 to 80 million years old [15,16]. Although there is some controversy about the results of these studies, reanalysis by another group of the *Tyrannosaurus rex* sample has led to analogous results [17]. As indicating by Bern *et al.* [17], contamination remains a tricky and possibly unresolvable issue for these ancient fossilized samples, necessitating considerable precautions during the extraction process.

## 2. Fibrillar Collagen Family

To better understand the evolution of fibrillar collagens, we will first describe some sequence particularities of these proteins by taking into account human data. In humans, the fibrillar collagen family included types I–III, V and XI, but also the more recently characterized types XXIV and XXVII [2,18–20]. As illustrated in Figure 1B, types XXIV and XXVII share some particularities with the other fibrillar collagen chains. Their major triple helix is slightly shorter (997 instead of 1014 to 1020 residues) and presents two successive glycine substitutions and one Gly-Xaa-Yaa-Zaa imperfection. Moreover, their *N*-propeptide domains are devoid of a minor triple-helical region, unlike the other fibrillar collagens. Human fibrillar α chains harbor different non-collagenous modules in their *N*-propeptide domains (Figures 1B and 1C). The proα1(I), proα1(II), proα1(III) and proα2(V) (e.g., a proα2(I) collagen polypeptide corresponds to the proα chain 2 of type I collagen) chains contain a VWC module in their *N*-propeptides while a TSPN domain is observed for the proα1(V), proα3(V), proα1(XI), proα2(XI), proα1(XXIV) and proα1(XXVII) chains. For the proα2(I) chain, the *N*-propeptide is almost entirely made of a short triple helical region. At the molecular level, types II and III collagens are made up of three identical chains (homotrimer). Structural considerations and tissue localizations suggest that collagens XXIV and XXVII form homotrimeric molecules [18–20]. Type I is generally an heterotrimer comprising two proα1(I) and one proα2(I) chains (Figure 1A), but homotrimeric molecules made of three proα1(I) chains have been detected. The situation of types V and XI is more complicated. These often represent heterotrimeric molecules, but a homotrimer of the proα1(V) chain has been characterized and composite molecules of types V and XI have also been described [21]. It should been noted that the proα3(XI) chain appears to be a modified product of the gene encoding the type II collagen chain [22,23].

**Figure 2.**
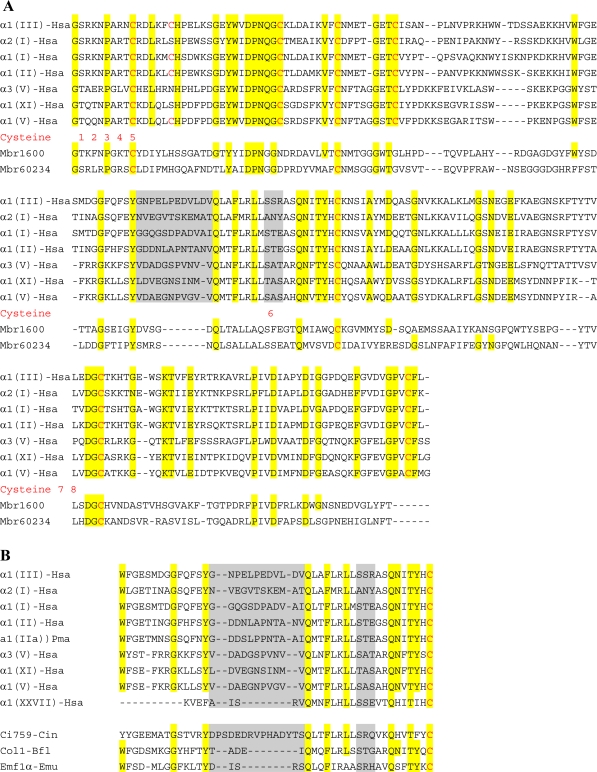
Multiple alignments of *C*-propeptide (COLF1) domains. (**A**) Selected human (Hsa) and choanoflagellate (Mbr, *Monosiga brevicolis*) COLF1 domains are aligned. Residues that are perfectly conserved among these proteins are shaded in yellow. Cysteine residues are colored in red and numbered. The grey boxes represent the chain selectivity recognition domains identified by Lees *et al*. [24]. (**B**) Chain recognition sequences in Metazoa. This sequence alignment is based on previous studies [12,25].

Since the same cell can synthesize different types of fibrillar collagen at the same time, a specific molecular mechanism is required for the recognition and discrimination of α chains during assembly of the procollagen molecule. Using chimeric recombinants of human proα2(I) and proα1(III) chains, Lee *et al*. [24] have characterized a discontinuous region of 15 amino acids in the *C*-propeptide which is involved in such recognition of fibrillar procollagen chains. Multiple alignment analysis of mammalian *C*-propeptides permitted these authors to show that this region corresponds to two relatively hydrophilic stretches of 12 and three amino acids separated by a highly conserved and hydrophobic sequence (Figure 2A). Hence, the *C*-propeptide plays a fundamental role allowing for cell type-specific assembly of fibrillar procollagen molecules.

## 3. The *N*-Propeptide Region of Fibrillar Procollagens

As indicated above, after removal of the propeptides from procollagens, the resultant collagen molecules are involved in the formation of striated fibrils. Once more, this is not a simple situation. Fibrils are generally heterotypic structures, being composed of one or two quantitatively major collagens (I–III) as well as one quantitatively minor collagen (V or XI). We can also distinguish fibrils present in cartilage (including types II and XI) from those in non-cartilage tissues (types I, III and V). Moreover, partial processing of the *N*-propeptide of minor fibrillar collagens in these heterotypic fibrils has been demonstrated. The structural importance of the retention of the *N*-propeptide in fibrils has been pointed out in several studies [26,27]. From the model of Linsenmayer *et al.* [27], the presence of the type V *N*-propeptide on the fibril surface regulates fibril diameter by sterically preventing the further addition of collagen molecules. In other words, the thinnest heterotypic fibrils have the highest minor collagen content. In the case of type XXVII collagen, it was recently shown that this unusual fibrillar collagen is involved in the formation of ultra-thin 10 nm thick non-striated fibrils [28]. However, another group has indicated that type XXVII is a component of non-banded fibrous structures, filamentous networks, and thin banded fibrils [29].

Four *N*-propeptide configurations have been described in human fibrillar collagen chains (Figure 1B). As shown in Figure 1C, most metazoan α chains can be assigned to one of these four types of *N*-propeptide. One exception to this rule occurs in Cnidaria, where proα chains possess an *N*-propeptide made of WAP or WAP and VWA modules (not shown in Figure 1B) in addition to a minor triple helix. Also not shown is the WASP module (eight cysteine residues), which we have demonstrated is similar to the VWC domain (10 cysteine residues) in terms of length, location in the *N*-propeptide and presence of two successive cysteine residues near the *C*-terminus [30]. The second exception concerns the presence in some sea urchin α chains of a series of a four-cysteine modules (called SURF modules) between a VWC domain and the minor triple helix within their *N*-propeptide regions (Figure 1C) [31,32].

**Figure 3.**
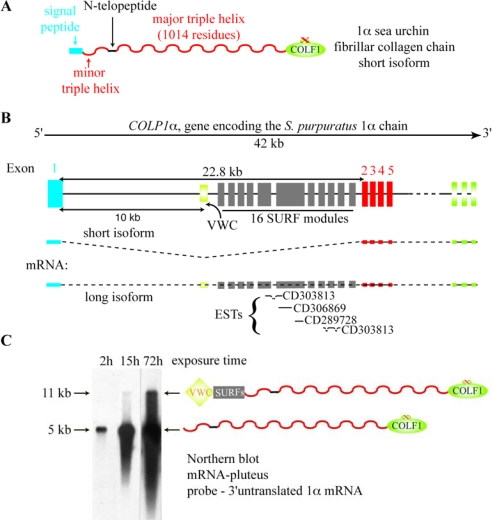
The sea urchin 1α fibrillar collagen chain. (**A**) Modular structure of the 1α chain. Domains are illustrated as in Figure 1. (**B**) the exon/intron structure of *COLP*1α, the gene encoding the *S. purpuratus* 1α chain. The *COLP1α* sequence is available using a sea urchin server (http://genome.ucsc.edu/cgi-bin/hgGateway; Scaffold69286:454253-495522 bp). Exons are represented by closed boxes. Below the *COLP1α* gene, the short and putative long mRNA isoforms are schematized. The four ESTs specific to the long 1α mRNA isoform are indicated with their accession number. (**C**) Northern-blot analysis of 1α mRNA. A short-exposure time of this Northern-blot was shown previously [34]. Here, we present different times of autoradiography, from 2 h to 72 h.

Another sea urchin α chain from *Strongylocentrotus purpuratus*, termed 1α, has an *N*-propeptide reduced to a minor triple helix reminiscent of the situation found in the vertebrate proα2(I) chain [33]. However, the large intronic sequence (approximately 22,800 bp) between the two first 5’ exons of the gene encoding the 1α chain might potentially encode one VWC and 16 SURF modules without perturbing the open reading frame (Figure 3). Blast analysis reveals that four ESTs (from *S. purpuratus* larva tissues) span part of these newly deduced exons (Figure 3). It should be noted that the study concerning the *S. purpuratus* 1α chain was carried out using blastula to pluteus embryos and that in all the cDNAs analyzed the open reading frame encoded a 1α chain presenting an *N*-propeptide reduced to the minor triple helix [33]. In agreement with the cDNA study, Northern-blot analysis revealed a 5 kb mRNA specific to the 1α chain. Interestingly, and as shown in Figure 3, over-exposed autoradiograms show an 11 kb mRNA band at the pluteus stage that might potentially encode the long isoform of the 1α chain.

## 4. The *C*-Propeptide of Fibrillar Collagens

The *C*-propeptide or COLF1 domain contains highly conserved sequences that are probably involved in its own structure, but is also punctuated by less conserved regions like those involved in chain selection [24,35]. The most important result concerning the evolution of this domain has been to realize that most of the defined chain selectivity recognition sequence is absent in invertebrate fibrillar collagens [36]. As shown in Figure 2B, only one invertebrate chordate α chain, Ci759-Cin, possesses a complete sequence. The relevance of this situation in regard to fibrillar collagen evolution will be discussed later. In spite of the increase of metazoan data, the COLF1 domain has only been described to date at the *C*-terminus of fibrillar collagen chains. Choanoflagellates are the closest living relatives of the Metazoa. Interestingly, King *et al.* [5] have indicated that the genome of the choanoflagellate *Monosiga brevicolis* can potentially encode two proteins including a triple-helical sequence and three possessing a COLF1 domain. Multiple alignment analysis (Figure 2A) reveals that *M. brevicolis* COLF1 modules lack Cysteine residues 2, 3, 5 and 8. In fibrillar collagens, Cys-5 and Cys-8 form an intra-chain disulfide bond while either Cys-2 or Cys-3 or both of them can be absent.

## 5. The Major Triple Helical Sequences and the Formation of an Ancestral Gene

**Figure 4.**
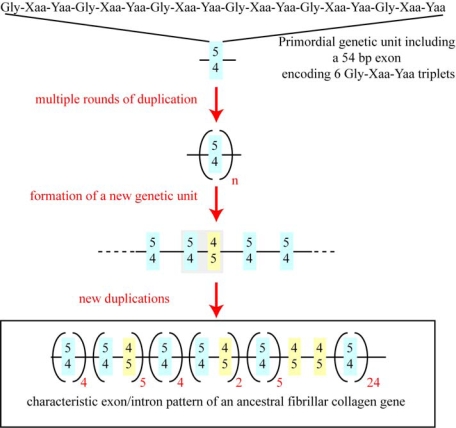
Hypothetical steps leading to the formation of an ancestral fibrillar collagen gene.

The characterization of the genes encoding fibrillar collagen chains have led to proposals concerning the exon-intron organization of an ancestral fibrillar collagen chain in addition to some steps leading to its formation. By chronological order, it was first obvious that half the exons encoding the major triple helix of types I and III collagens were 54 bp of length while the others are multiples of 54 bp (108 and 162 bp) or multiples of 54 bp minus 9 bp (45 and 99 bp). From this observation, Yamada *et al*. [37] suggested that the primordial genetic unit of an ancestral fibrillar collagen includes an exon of 54 bp in length, beginning with an intact Glycine codon, ending with an intact Yaa codon, and encoding six Gly-Xaa-Yaa triplets. From this point of view, the formation of a putative ancestral gene arose from the multiple duplications of this primordial unit. The presence of the 45 bp and 99 bp exons in fibrillar collagen genes could be explained in this hypothesis by unequal crossing-over. Later on, from the study of a freshwater sponge fibrillar collagen gene and by comparing this data with the exon-intron structures of mammalian types I–III genes, we have been able to propose the exon-intron organization of a putative ancestral fibrillar collagen gene [38]. In comparison to the Yamada model, we suggest that two genetic units are at the origin of the fibrillar collagen genes. As proposed by Yamada *et al.* [37], the first steps have been multiple rounds of duplication of an exon of 54 bp. An unequal crossing-over event led to the formation of a 45 bp exon beginning by an intact Glycine codon. As shown in Figure 4, multiple duplications of a new genetic unit including a 54 bp and a 45 bp exon might explain the particular distribution of these two types of exons in fibrillar collagen genes. Interestingly, and with the availability of more genomic data, the exon-intron organization of numerous genes [25,30,39,40] is consistent with that suggested for an ancestral fibrillar collagen gene ([41] and Figure 4).

## 6. Using Triple Helical Sequences to Decipher Fibrillar Collagen Evolution

Preceding phylogenetic analyses, molecular and electron microscopic studies have suggested that fibrillar collagens from invertebrates, and more especially from diploblast animals seem to be related to the vertebrates types V/XI [9,42,43]. Hence, in sponges, striated fibrils have a uniform diameter of 25 nm, a situation observed in vertebrates for heterofibrils containing types V or XI collagens. A second observation allowing the first classification of vertebrate fibrillar collagens has resulted from the sequencing of the related genes. Takahara *et al*. [39] proposed, from the exon/intron distribution in the region encoding the major triple helix that the fibrillar collagens could be divided into two subgroups. The first includes the genes encoding types I–III and the proα2(V) chains while the second includes those encoding the proα1(V), proα1(XI) and proα2(XI) chains [39,40]. Later on, sequencing projects demonstrated that *COL5A3* is a member of the second group. The next step in knowledge of the evolution of this collagen family was to make a phylogenetic analysis using human α chains and a few invertebrate fibrillar collagens [44]. Despite the small number of sequences used, it was clear that vertebrate α chains could be divided in two subfamilies, this study confirming the previous suggestion made from simple observations of gene organization. Hence, the first (types I–III, proα2(V) chain) and second (proα1(V), proα3(V), proα1(XI), and proα2(XI) chains) subfamilies were called the A and B clades, respectively. In agreement with these studies, the *N*-propeptide composition of the A and B chains are different. A clade members possess a VWC module in their N-propeptide, while B clade members possess a TSPN module. It should be noted that the proα2(I) chain lacks the VWC domain, but is included in the A clade. More recently, a third subfamily of fibrillar collagen that includes the proα1(XXIV) and proα1(XXVII) chains was characterized, and called the C clade [19]. While at this point the evolutionary relationship of vertebrate fibrillar collagen chains was well understood, there remained some difficulty and/or controversy in assigning invertebrate fibrillar α chains to one of the vertebrate clades despite the presence in several invertebrate α chains of a VWC module in their *N*-propeptide. For these phylogenetic analyses [19,44], the authors used the *C*-propeptide sequences. However, due to several factors (variability in length and sequence), this domain is not sufficiently informative to decipher the evolution of the fibrillar collagens. A new approach was to postulate that the conservation of the exon/intron organization of metazoan fibrillar collagen genes in the region encoding the major triple helix reflects conservation of related amino acid sequences [25]. In agreement with this methodological approach, it should be noted that, with the availability of complete sequences of eukaryotic genomes, exon-intron-structures have indeed been used as a novel source of evolutionary information [45,46]. As indicated by Csurös *et al*. [47], “comparative-genomic studies show that numerous intron positions in orthologous genes are conserved at great evolutionary depths, for example, between plants and animals”. Hence, the use of intron positions might improve multiple protein sequence alignments in regions of questionable alignment [48]. It is even possible to align two or more unrelated collagenous sequences by this method, while multiple alignments generally result in numerous gaps. In contrast, multiple alignments of bilaterian major triple helices confirm the pattern of introns of fibrillar collagen genes [25,30].

**Figure 5.**
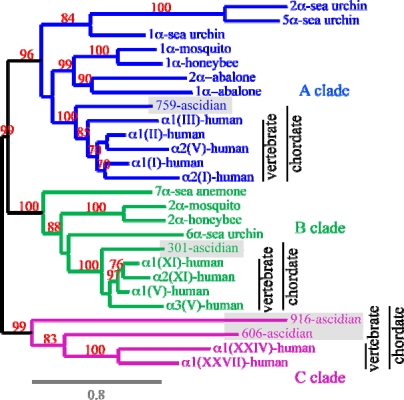
Phylogenetic analysis of some metazoan fibrillar collagen chains. This unrooted tree was modified from a previous study [30]. The illustration was drawn using the TreeDyn program [49]. Gray boxes indicate the ascidian fibrillar collagen chains.

**Figure 6.**
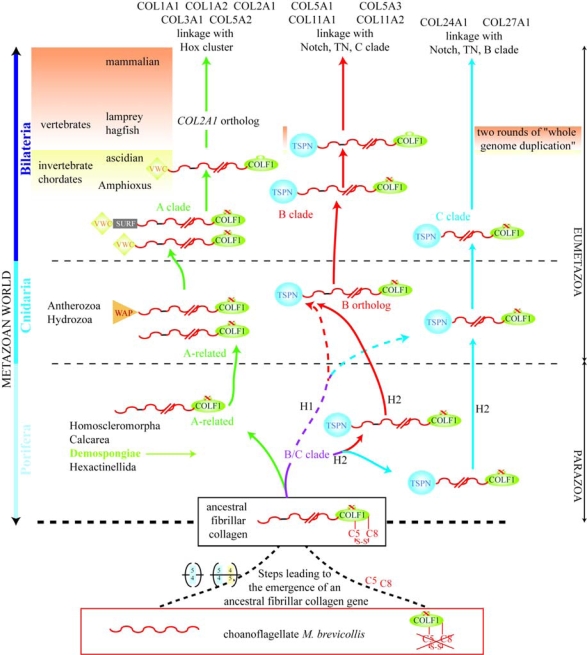
Evolution of the fibrillar collagen family. This model was modified from a previous study [30]. It should be followed from the bottom to the top. In this model, we suggest that the steps leading to the formation of an ancestral α chain occurred before the metazoan radiation and the divergence of the three clades predated the Parazoa-Eumetazoa split. The different steps (emergence of the different clades, module or chain selection sequence acquisition) are presented. We have two major hypotheses concerning the B/C clades. In the H1 scenario, sponges possess fibrillar collagen chains containing a TSPN module and the related genes have duplicated before the divergence of the B and the C clades from an ancestral B/C clade gene. In this hypothesis, the emergence of the B and C clades occurred between Parazoa-Eumetazoa and Cnidaria-Bilateria split. In hypothesis H2, B and C clade genes are present in demosponges and their emergence predated metazoan cladogenesis.

Using triple-helical sequence with or without the *C*-propeptide domain, it was possible to investigate more precisely the fibrillar collagen story (Figures 5 and 6) Altogether, these studies have permitted the evolution of this protein family to be followed from sponges to humans [12,19,25,30,50,51]. As shown in Figure 5, the three fibrillar collagen clades, defined in humans have emerged before the emergence of chordates. Hence, the invertebrate chordate *Ciona intestinalis* (ascidian) clearly possesses a member of each clade, but also another α chain (906-ascidian) related to the C clade and presenting numerous imperfections and Glycine substitutions in its major triple helical domain. With the availability of poriferan (parazoan) and cnidarian (Radiata, eumetazoan) genomes, new analyses have revealed the early evolution of the fibrillar collagen family [30,50]. Hence, there is strong phylogenetic support for the hypothesis that the emergence of the A, B and C clades predated the Radiata–Bilateria split [30]. Moreover, and although not strongly supported by phylogenetic analyses, there is compelling evidence that the emergence of the three clades predated the divergence of poriferan lineages. Hence, the modular structures of the sponge fibrillar collagen chains are in good agreement with this hypothesis.

## 7. Suggested Evolutionary Model for the Fibrillar Collagen Family

**Figure 7.**
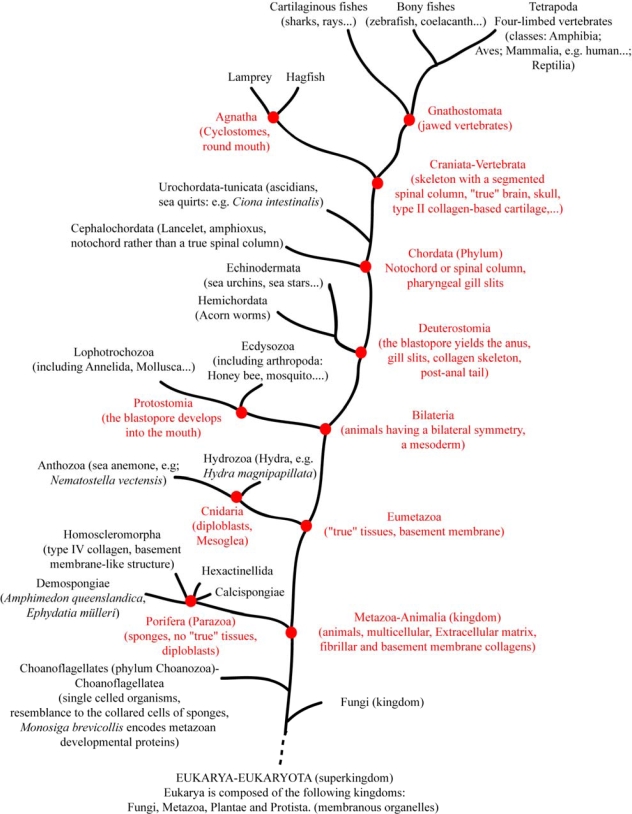
Simplified Eukarya tree of life with a special focus on the kingdom Animalia. Most of the animals discussed in the text are represented. This tree is based on several studies [51–58]. Sponges are presented as a monophyletic group [57].

A model for the evolution of fibrillar collagens is presented in Figure 6. Moreover, to better understand the Figure 6, a simplified tree of life is illustrated in Figure 7. From the literature available to date, fibrillar collagens seem to be specific to Metazoa. Although we could not reject the hypothesis that fibrillar collagen information was lost in Choanoflagellatea (the sister group of Metazoa, Figure 7), several data favor the idea that an ancestral fibrillar collagen gene arose in the lineage leading up to the Metazoa. Hence, the choanoflagellate *M. brevicollis* might potentially encode proteins including either triple helical or COLF1 sequences while fibrillar collagens are present in sponges. The three *M. brevicollis* COLF1 sequences lack cysteine residues 5 and 8. The fact that cysteine residues 5 and 8 form an intra-chain disulfide bond and are strictly conserved among fibrillar collagen chains suggests that they play an important function in the structure of the COLF1 module. As shown in Figure 6, the COLF1 domain present in the ancestral fibrillar collagen chain might possess cysteine residues 5 and 8. The lack of cysteine residues 2 and 3 in *M. brevicollis* COLF1 sequences is more questionable. Hence, previous studies have suggested that only α chains possessing all 8 cysteine residues are able to form homotrimeric procollagen molecules [59,60]. In contrast, these two cysteine residues are absent in some invertebrate fibrillar collagen chains [30]. Moreover, recombinant studies using semi-intact cells have shown that cysteine residue 2 is not required during chain association and triple helix folding [61].

The emergence of the A and B/C clades occurred before the parazoan–eumetazoan split, the earliest divergence among extant animal phyla. As shown in this model (Figure 6), the divergence between the ancestral B and C clade fibrillar collagen chains occurred either before the separation of poriferan and eumetazoan lineages (H1 hypothesis) or predated metazoan cladogenesis (H2). The modular organization of sponge and sea anemone fibrillar α chains (Figure 1C and see Figure 1 in Reference [30]) is in favor of the H2 hypothesis, and reveals that only B clade collagens have conserved their *N*-propeptides and triple helix characteristics from sponges to humans. For the A clade, we can note that the *N*-propeptide seems to be reduced to the minor triple helix in sponge. The formation of an A clade fibrillar collagen chain possessing a VWC module in its *N*-propeptide predated the bilaterian radiation. The VWC module might have evolved from a WAP module present in Cnidaria as previously suggested [30], or the presence of WAP sequences in A clade related α chains might be specific to Cnidaria. The C clade is the less known family, and in the absence of more sequence data, we can only indicate that the major triple helix of C clade members seems to have evolved more rapidly than the comparable domains in clades A and B. Hence, the ascidian C clade fibrillar collagen chains do not have the Gly-Xaa-Yaa-Zaa imperfection present in types XXIV and XXVII.

As indicated in Figure 6, lamprey and hagfish (agnathans) have true orthologs of *COL2A1*, the gene encoding the vertebrate type II collagen (member of the A clade), which is the major structural protein of cartilage in gnathostomes [62–64]. Although the cartilage of agnathans was first described as a non-collagenous tissue [65], the presence of type II collagen has been demonstrated, suggesting that the formation of a collagen-based cartilage predated the agnathan-gnathostome split. This hypothesis has to be related to recent studies indicating that the two rounds (2R) of whole genome duplication occurred between the origin of chordates and before the divergence between cyclostomes and gnathostomes [66,67]. Hence, Kuraku [67] suggests that “a post-2R state is a genomic synapomorphy for all extant vertebrates”. The relationships between fibrillar collagens and cartilage have also been investigated in invertebrates [12,51,63,68]. Rychel *et al*. [51] have shown that collagenous proteins are present in the pharyngeal cartilage of hemichordates and cephalochordates, this result suggesting that the formation of this collagenous tissues occurred near the time of deuterostome diversification [68]. In lancelet, the gene encoding this collagenous protein is expressed in the notochord [63]. It is related to the A clade and might be defined as the pre-2R ortholog of types I, II, III and the proα2(V) fibrillar collagen chains. In jawed vertebrates, the A clade genes are mostly expressed in notochord and/or notochordal sheath. As indicated by Zhang and Cohn [63], the vertebrate chondrocytes that express the type II gene may have evolved from notochordal cells.

The evolution of the chain selection sequence present in the COLF1 module (Figure 6) has been firstly described as a model of molecular incest [36]. In this model, a rare genomic event led to the formation of a sequence encoding a complete chain selection region at the dawn of the chordates and before the two whole genome duplication events. This gene might encode a fibrillar proα chain participating to the formation of a homotrimeric procollagen molecule. After the first round of duplications, the two newly formed genes can make the same homotrimeric molecule. With time, these two genes diverge but their translational product might still trimerize together in what is now an incestuous relationship. For the authors [36], this model could hold for all the multimeric proteins. Later on and from the studies of invertebrate chordate fibrillar collagens, another group pointed out that it is not one but two rare genomic events that preceded the two rounds of genome duplication [12]. The first event occurred before the ascidian-vertebrate split and permitted the formation of an A clade gene encoding a fibrillar collagen possessing a complete chain selection sequence. The second rare genomic event predated the vertebrate radiation and led to the formation of a B clade gene including the complete coding sequence of the chain selection domain. The functional importance of the chain selection sequence in vertebrates led us to think about the situation of invertebrate fibrillar collagen chains. Hence, seven and eight fibrillar collagen chains have been described in sponges and sea anemones, respectively [30]. All these α chains having an incomplete chain selection sequence, other mechanisms might be used in invertebrates. First and with the lack of biochemical and developmental data, we can imagine that these sponge or sea anemone genes are differentially expressed in these organisms. Second, some of these α chains might be indiscriminately used in the formation of heterotrimeric molecules. Thirdly, other variable regions of the COLF1 domain might be involved in the chain selection in invertebrates.

## 8. Conclusions

During the last few years, data arising from sequencing projects have led to a better understand the evolution of the fibrillar collagen genes [30,50], and have highlighted the importance of the chain selection sequence and of the two rounds of genome duplication in the evolution of vertebrate development [53,62,63]. Another level of complexity concerns the molecular mechanisms leading to the formation and structural aspects of collagen fibrils. In vertebrates, fibrils are generally heterotypic, their diameters depending on the procollagen types present and their ratio, as well as the *N*-propeptide maturation of minor procollagens and interactions with other extracellular matrix components [69]. Moreover, it has been suggested that the minor fibrillar collagens (types V and XI) play a pivotal function in the nucleation of fibril assembly [69–71], and have been named as the “nucleators” of the initiation of the collagen fibrillogenesis [69]. Interestingly, the B clade fibrillar collagen chains have conserved the same modular organization and triple helix characteristics from sponges to humans. While little is known about the composition of invertebrate fibrils, we have previously demonstrated in sea urchin the presence of heterotypic fibrils made of quantitatively major and minor collagen molecules undergoing distinct maturation of their *N*-propeptide domains [32]. In sponges, all collagen fibrils have a thin, uniform diameter of 20–25 nm [2], although phylogenetic studies suggest that members of the three fibrillar collagen clades are present in these animals. During the next years, a new challenge will be to decipher the evolution of the collagen fibrils.
